# Facial Soft Tissue Characteristics Among Sagittal and Vertical Skeletal Patterns: A Cone-Beam Computed Tomography Study

**DOI:** 10.7759/cureus.44428

**Published:** 2023-08-31

**Authors:** Nora Alhazmi, Faris Alrasheed, Khalid Alshayea, Talal Almubarak, Bandir Alzeer, Meshal s Alorf, Abdulaziz Alshanqiti, Maram Albalawi

**Affiliations:** 1 Preventive Dental Science, King Saud Bin Abdulaziz University for Health Sciences, Riyadh, SAU; 2 Research and Development, King Abdullah International Medical Research Center, Riyadh, SAU; 3 Dental Hospital, Ministry of National Guard for Health Affairs, Riyadh, SAU; 4 College of Dentistry, King Saud Bin Abdulaziz University for Health Sciences, Riyadh, SAU; 5 Biostatistics, King Abdullah International Medical Research Center, Riyadh, SAU

**Keywords:** orthodontics, vertical, sagittal, cbct, soft tissue

## Abstract

Background

Facial esthetics depend on the skeletal and dental structures underlying variable facial soft tissue thickness. In this social context, determining the relationship between external soft tissue and underlying skeletal and dental hard tissue is essential for detailed orthodontic diagnosis and treatment planning.

Objective

This study aims to measure facial soft tissue thickness in different sagittal and vertical skeletal patterns.

Methodology

This is an observational study utilizing pre-existing cone-beam computed tomography (CBCT) images of 170 subjects (110 females and 60 males) with a mean age group of 37.45 ± 13.83 years. CBCT images were then classified sagittally based on the point A-Nasion-point B (ANB) angle from Steiner's analysis into skeletal Class I, Class II, and Class III. Furthermore, vertical patterns were grouped based on the Frankfort-mandibular plane angle (FMA) from Tweed's analysis into hyperdivergent, hypodivergent, and normodivergent facial types. One-way ANOVA was used to compare the means of facial soft tissue thickness between the skeletal groups, followed by Tukey's post-hoc test for individual comparison. Multinomial logistic regression analysis was used to test the association between gender, age, and skeletal groups. The significance level was 0.05.

Results

One-way ANOVA revealed statistically significant differences in both sagittal and vertical groups (p≤0.05). Tukey's post hoc analysis showed that the skeletal Class III group has increased soft tissue thickness in the subnasale, upper lip, and mention compared to Class I and Class II subjects. Moreover, the hypodivergent group demonstrated increased soft tissue thickness in gnathion and mentioned landmarks in relation to the other groups. Multinomial logistic regression analysis showed significant differences between groups according to both gender and sagittal skeleton patterns (p≤0.05), with males less likely to be in Class II.

Conclusions

Skeletal Class III and hypodivergent groups have thicker soft tissue in specific facial landmarks. Sexual dimorphism was marked in soft tissue measurements.

## Introduction

The shape and size of the head and face, known as craniofacial morphology, is influenced by genetics and the environment. These factors work together to create the unique characteristics of an individual's face [1]. The impact of genetics and the environment can lead to a wide range of facial profiles within a population [1]. Facial harmony and attractiveness are associated with social acceptance, success, and psychosocial well-being [2, 3]. Yet, facial proportions and esthetics depend on the skeletal and dental structures underlying variable facial soft tissue thickness [4]. For instance, increased lip thickness is perceived as preferable in females and might indicate youth in modern women, while less lip thickness is more desirable in males [5, 6]. In this social context, determining the relationship between external soft tissue and underlying skeletal and dental hard tissue is essential for detailed orthodontic diagnosis and treatment planning [4, 7]. It is, therefore, important for the orthodontist to be able to analyze and predict the soft tissue characteristics in different skeletal patterns.

The anterior-posterior skeletal malocclusions have a significant impact on the facial soft tissue, leading to discrepancies in the soft tissue dimensions [6, 8]. Each skeletal malocclusion has specific soft tissue characteristics representing its hard tissue counterpart [8]. For example, significant statistical differences exist between the soft tissue measurements in Class ll and Class III malocclusion, and skeletal Class III has been reported to have significantly thicker soft tissue in the maxilla among Brazilian adults [9]. Analysis of soft tissue dimensions in different sagittal dimensions can, therefore, be used as a diagnostic aid in orthodontic practice [7, 9].

Vertical skeletal relationships are another critical aspect of facial soft tissue affecting facial attractiveness. According to the literature, attractiveness scores reduce as the vertical proportions diverge from normal [10]. Vertical facial patterns are classified into three different patterns: high angle (hyperdivergent), low angle (hypodivergent), and normal angle (normodivergent) [11]. To the authors' knowledge, there is a lack of literature analyzing soft tissue thickness in different vertical skeletal patterns. In addition, most existing studies have been conducted with lateral cephalometric images [12]. Two-dimensional (2D) cephalometrics suffers from drawbacks such as inaccurate landmark locations due to superimposition of structures, image distortion, and magnification errors; however, better results can be attained using 3D cone beam computed tomography (CBCT) [13]. Indeed, CBCT images are increasingly being utilized in therapeutic settings to show complex facial features made up of both hard and soft tissues in three dimensions [14, 15]. To the authors' knowledge, this is the first orthodontic study to combine anterior-posterior and vertical skeletal patterns in quantifying soft tissue thickness in the Saudi Arabian population. Previous studies reported significant ethnic variations in facial soft tissue thickness among different population groups [16, 17]. In addition, these studies indicate that what is considered normal for one group must not be considered normal for other ethnic and racial groups. Hence, it is crucial to treat each racial group based on its own characteristics [18]. For that reason, this study focused on the Saudi Arabian population to develop individual standards for its ethnic group. It is important to point out that orthodontic diagnosis relies on facial soft tissue profile, and treatment outcome is evaluated and displayed by soft tissue appearance. This present study, therefore, aims to use 3D CBCT images to measure facial soft tissue thickness in different anterior-posterior skeletal patterns (skeletal Class I, Class II, and Class III) and different vertical skeletal patterns (normodivergent, hyperdivergent, hypodivergent). The null hypothesis was that there were no differences between the sagittal and vertical skeletal groups in terms of soft tissue thickness.

## Materials and methods

Our study was conducted using pre-existing CBCTs taken for dental or surgical purposes between 2017 and 2023. The radiographs were randomly selected from the archive of the King Abdulaziz Medical City in Riyadh (KAMC-RD) and the College of Dentistry (COD) in King Saud bin Abdulaziz University for Health Sciences (KSAU-HS). The study is a retrospective, cross-sectional, and comparative study that was approved by the institutional board of King Abdullah International Medical Research Center (KAIMRC) (protocol number: NRC22R/302/06, date of approval August 22nd, 2022). The inclusion criteria were that the CBCT images must be of decent quality and of subjects 18 years and above and Saudi Arabian. The variables were measured using CBCT imaging software (Romexis software version 4.5, Planmeca OY, Helsinki, Finland) with a volume size of 23.0 × 17.3 cm or above and a voxel size of 400 µm. Subjects with previous orthodontic treatment, a trauma in the head and neck region, congenital abnormalities, cleft lip and palate, previous plastic surgery, or previous orthognathic surgery were excluded, as were CBCT images with distortions.

Eligible CBCT images were then grouped by one examiner (NA) and divided into sagittal groups based on Steiner's point A-Nasion-point B (ANB) angle analysis [19]: group 1 was skeletal Class I pattern (1≤ ANB ≤4); group 2 was skeletal Class II pattern (ANB>4); and group 3 was skeletal Class III pattern (ANB≤0).

In addition, the same examiner (NA) classified the vertical groups based on Tweed's Frankfort-mandibular plane angle (FMA) analysis [11]: group 1 was hyperdivergent skeletal pattern (FMA>30); group 2 was hypodivergent skeletal pattern (FMA<20); and group 3 was normodivergent skeletal pattern (20< FMA <30).

**Figure 1:**
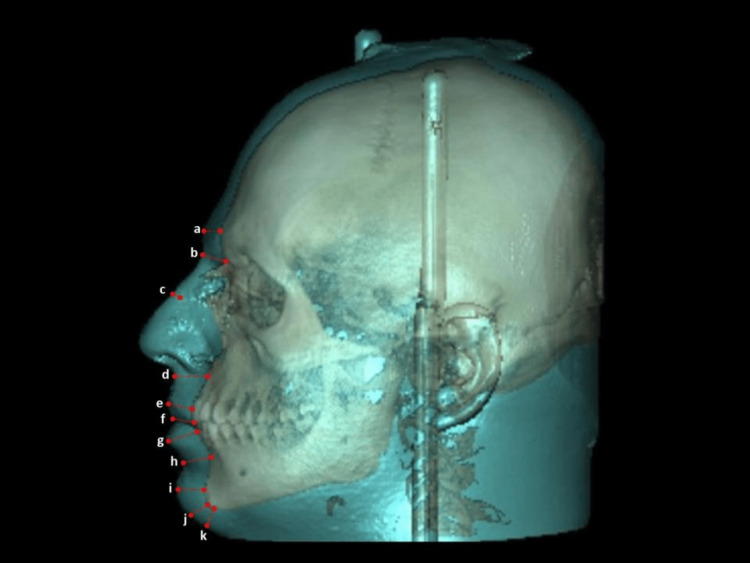
Definitions and measurements of CBCT landmarks in sagittal view (a) hard tissue glabella - soft tissue glabella (GL_h_-GL_s_), (b) hard tissue nasion - soft tissue nasion (N_h_-N_s_), (c) hard tissue rhinion - soft tissue rhinion (Rhi_h_-Rhi_s_), (d) hard tissue subspinale - soft tissue subnasale (Ss_h_-Sn_s_), (e) maxillary incisor - labrale superius (U1-LS_s_), (f) hard tissue incision - soft tissue stomion (Inc_h_-Sto_s_), (g) mandibular incisor - labrale inferius (L1-Li_s_), (h) hard tissue supramentale - soft tissue supramentale (Sm_h_-Sm_s_), (i) hard tissue pogonion - soft tissue pogonion (Pg_h_-Pg_s_), (j) hard tissue gnathion - soft tissue gnathion (Gn_h_-Gn_s_), (k) hard tissue menton - soft tissue menton (Me_h_-Me_s_) CBCT - cone-beam computed tomography

Thereafter, the examiner (NA) was blinded to the groups through the creation of a new Excel spreadsheet (Microsoft, Redmond, Washington) by another examiner (FA) with subjects coded and lacking the skeletal groups. Then, the facial soft tissue thickness was assessed across eleven different landmarks as described by Gomes et al. [9] (Figure 1). The landmarks were defined in the sagittal, coronal, and axial views, as shown in Table 1. The total sample size was 170 subjects (110 females and 60 males) with a mean age of 37.45 ± 13.83 years. The sample distribution per skeletal group, as well as the gender distribution, is illustrated in Table 2.

**Table 1:** Definition of landmarks in different CBCT views: sagittal, coronal, and axial CBCT - cone-beam computed tomography

Landmark (linear mm)	Sagittal view	Coronal view	Axial view
Hard tissue glabella - soft tissue glabella (GL_h_-GL_s_)	Linear distance in millimeters from the most prominent part of frontal in the midsagittal bone to the most anterior soft tissue of the forehead	Superior to the nasal bone and medial to the orbital rims in the midline of the frontal bar to the most anterior soft tissue	Linear distance in millimeters from the most prominent part in the midline of the frontal bone to the most anterior soft tissue forehead
Hard tissue nasion - soft tissue nasion (N_h_-N_s_)	Linear distance in millimeters from the nasofrontal suture to the point directly anterior to the nasofrontal suture in the midline	Inferior to the frontal bar superior to the nasal bone and with soft tissue directly anterior to it	The most anterior superior bone in the midline of the nasal bone to the soft tissue directly anterior to it
Hard tissue rhinion - soft tissue rhinion (Rhi_h_-Rhi_s_)	Distance from the most rostral (end) point on the internasal suture to the corresponding soft tissue	The lower border of the internasal suture and the soft tissue anterior to it	Inferior to the nasion, superior to the nasal tip, medial to the ala of the nose, and to the soft tissue directly anterior to it
Hard tissue subspinale - soft tissue subnasale (Ss_h_-Sn_s_)	Inferior to the nasal spine superior to incision supe to the soft tissue directly anterior to it	Inferior to the nasal bone superior to the incisor	From the anterior portion of the ala of the nose to the deepest point of the nasal spine
Maxillary incisor - labrale superius (U1-Ls)	Upper lip thickness; linear distance in millimeters from the most prominent labial point of maxillary incisor to the labrale superius	The most inferior part of the maxillary incisor to the point inferior to the philtrum and superior to the lower lip	Medial to the lateral maxillary incisor to the point medial to the commissures
Hard tissue Incision - soft tissue stomion (Inc_h_-Sto_s _)	From the upper incisor posterior to the dorsal of the lips	From the upper incisor posterior to the dorsal of the lips	From the incisal tip of the upper central incisor to the mid-point in the termination point of the upper lip
Mandibular incisor - labrale inferius (L1-Li)	Lower lip thickness; linear distance in millimeters from the most prominent labial point of the mandibular incisor to the labrale inferius	The most superior part of the mandibular incisor and superior to the mentolabial fold	Medial to the lateral mandibular incisor to the point medial to the commissures
Hard tissue supramentale - soft tissue supramentale (Sm_h_-Sm_s_)	Linear distance in millimeters Distance from orthodontic point B and the supramentale’ (deepest midline point of the mentolabial sulcus)	Superior to the pogonion, inferior to the mandibular incisor and to the soft tissue directly anterior to it	Medial to the mental foramen in the midline of the mandible to the soft tissue directly anterior to it
Hard tissue pogonion - soft tissue pogonion (Pg_h_-Pg_s_)	Linear distance in millimeters from the most anterior median point on the mental eminence of the mandible to the most anterior midpoint of the chin, located on the skin surface anterior to the identical bony landmark	The most prominent point of the mandible to the soft tissue directly anterior to it	in the midline of the mandible to the soft tissue directly anterior to it
Hard tissue gnathion - soft tissue gnathion (Gn_h_-Gn_s_)	Linear distance in millimeters between the most inferior median point of the mandibular symphysis and the most inferior median point of the chin.	The most prominent inferior part of the mandible to the soft tissue directly anterior to it	In the midline of the mandible to soft tissue directly anterior to it
Hard tissue menton - soft tissue menton (Me_h_-Me_s_)	Linear distance in millimeters between the most inferior point on the contour of the mandibular symphysis and the soft tissue of the inferior point on the contour of the mandibular symphysis	The most inferior part of the mandible to the soft tissue directly anterior to it	In the midline of the mandible to soft tissue directly anterior to it

**Table 2:** Distribution of subjects in different skeletal patterns

Skeletal patterns		N	Percentage (%)	Female	Male
Sagittal groups	Class I	74.00	43.53	43.00	31.00
	Class II	63.00	37.06	51.00	12.00
	Class III	33.00	19.41	16.00	17.00
	Total	170.00	100.00	110.00	60.00
Vertical groups	Normodivergent	85.00	50.00	59.00	26.00
	Hypodivergent	39.00	22.94	19.00	20.00
	Hyperdivergent	46.00	27.06	32.00	14.00
	Total	170.00	100.00	110.00	60.00

The calculation of the sample size was performed using Number Cruncher Statistical System (NCSS) software 2022 version (NCSS LLC company, Kaysville, Utah) and the Power Analysis and Sample Size software (PASS) version 15 (NCSS LLC company, Kaysville, Utah), based on the effect size stated in Gomes et al. study [9]. A minimum sample size of 11 subjects per sagittal skeletal group achieves 80% power to detect differences among the means versus the alternative of equal means using an F test with a 0.05 significance level. As for the vertical skeletal groups, a total sample of 60 subjects (20 subjects per group) was adequate to obtain 80% power and a type I error of 5%.

The Shapiro-Wilk test was used to assess the normality of the dataset. A Chi-squared test was performed to compare males and females in relation to facial soft tissue thickness. Furthermore, the one-way ANOVA test was used to investigate the mean differences in the soft tissue thickness between the skeletal sagittal and vertical groups, followed by Tukey's post hoc test for individual comparison. Moreover, multivariant logistic regression analysis was used to evaluate the relationship between age, gender, and soft tissue thickness, with p-values less than .05 considered statistically significant.

For intra-examiner reliability, twenty CBCT images were chosen randomly one month after the first analysis and were re-measured by the same orthodontist (NA). The intraclass correlation coefficient (ICC) test was used for intra-examiner reliability. Statistical Analysis Software (SAS) (version 9.4, SAS Institute Inc., Cary, North Carolina) was used for data analysis.

## Results

Since the Shapiro-Wilk test and histograms showed that the sample data was normally distributed, a parametric test (T-test) was used in the present study. This showed statistically significant differences between genders (p-value<0.05) in the majority of regions, except for Smh-Sms (Table 3). One-way ANOVA, revealed statistically significant differences between the three skeletal sagittal groups (p-value≤0.05). Tukey's post hoc analysis showed that, compared to Class I and Class II, the skeletal Class III group had increased facial soft tissue thickness in GL_h_-GL_s_ (p-value=0.0268), N_h_-N_s_ (p-value=0.0029), Rhi_h_-Rhi_s_ (p-value=0.0078), Ss_h_-Sn_s_ (p-value=0.0001), U1-LS (p-value≤0.0001), Inc_h_-Sto_s_ (p-value=0.0037) and Me_h_-Me_s_ (p-value=0.0024). Table 4 revealed more details about the differences between the three classes.

**Table 3:** Soft tissue thickness measurements in male and female subjects * indicates a p-value of <0.05. T-test was used to investigate the differences between means in genders. GL_h_-GL_s_: hard tissue glabella - soft tissue glabella, N_h_-N_s_: hard tissue nasion - soft tissue nasion, Rhi_h_ -Rhi_s_: hard tissue rhinion - soft tissue rhinion, Ss_h_ -Sn_s_: hard tissue subspinale - soft tissue subnasale, U1-LS: maxillary incisor - labrale superius, Inc_h_-Sto_s_: hard tissue incision - soft tissue stomion, L1-Li: mandibular incisor - labrale inferius, Sm_h_-Sm_s_: hard tissue supramentale - soft tissue supramentale, Pg_h_ -Pg_s_: hard tissue pogonion - soft tissue pogonion, Gn_h_ -Gn_s_: hard tissue gnathion - soft tissue gnathion, Me_h_ -Me_s_: hard tissue menton - soft tissue menton

Landmarks	Gender	Mean (mm)	Standard deviation (mm)	p-value
GL_h_-GL_s_	Male	5.74	1.20	0.0004*
	Female	5.06	1.14	
N_h_-N_s_	Male	7.32	1.58	<0.0001*
	Female	5.65	1.14	
Rhi_h_-Rhi_s_	Male	3.03	0.89	0.0003*
	Female	2.54	0.69	
Ss_h_-Sn_s_	Male	15.54	2.40	<0.0001*
	Female	13.29	1.54	
U1-LS	Male	11.95	2.10	<0.0001*
	Female	10.07	1.98	
Inc_h_-Sto_s_	Male	7.36	2.56	0.0012*
	Female	6.10	1.94	
L1-Li	Male	14.74	2.05	<0.0001*
	Female	12.68	2.37	
Sm_h_-Sm_s_	Male	12.11	1.92	0.4764
	Female	11.91	1.62	
Pg_h_-Pg_s_	Male	15.86	3.10	<0.0001*
	Female	14.02	2.72	
Gn_h_-Gn_s_	Male	5.75	1.69	0.0138*
	Female	5.11	1.56	
Me_h_-Me_S_	Male	5.39	1.65	0.0015*
	Female	4.61	1.43	

**Table 4:** Soft tissue thickness measurements in different sagittal skeletal groups One-way ANOVA showed significant differences in Class III group in specific landmarks: a) significant differences in Class I vs. Class II (p-value≤0.05) (Tukey's post hoc test), b) significant differences in Class II vs. Class III (p-value≤0.05) (Tukey's post hoc test), and c) significant differences in Class III vs. Class I (p-value≤0.05) (Tukey's post hoc test). GLh-GLs: hard tissue glabella - soft tissue glabella, Nh-Ns: hard tissue nasion - soft tissue nasion, Rhih -Rhis: hard tissue rhinion - soft tissue rhinion, Ssh -Sns: hard tissue subspinale - soft tissue subnasale, U1-LS: maxillary incisor - labrale superius, Inch-Stos: hard tissue incision - soft tissue stomion, L1-Li: mandibular incisor - labrale inferius, Smh-Sms: hard tissue supramentale - soft tissue supramentale, Pgh -Pgs: hard tissue pogonion - soft tissue pogonion, Gnh -Gns: hard tissue gnathion - soft tissue gnathion, Meh -Mes: hard tissue menton - soft tissue menton

Sagittal groups	N (total=170)	Variables	Mean (mm)	Standard deviation (mm)
Class I	74	GL_h_-GL_s_	5.30	1.08
		N_h_-N_s_	6.30	1.52
		Rhi_h_-Rhi_s_	2.81^ a^	0.82
		Ss_h_-Sn_s_	14.06^ c^	1.94
		U1-LS	11.07^ a^	2.34
		Inc_h_-Sto_s_	6.38^ c^	2.12
		L1-Li	13.52	2.29
		Sm_h_-Sm_s_	12.07	1.66
		Pg_h_-Pg_s_	14.72	2.93
		Gn_h_-Gn_s_	5.29	1.56
		Me_h_-Me_S_	4.84^ c^	1.61
Class II	63	GL_h_-GL_s_	5.06^ b^	1.14
		N_h_-N_s_	5.82^ b^	1.34
		Rhi_h_-Rhi_s_	2.47^ a,b^	0.68
		Ss_h_-Sn_s_	13.44^ b^	1.76
		U1-LS	9.76^ a,b^	1.94
		Inc_h_-Sto_s_	6.13^ b^	2.15
		L1-Li	13.06	2.78
		Sm_h_-Sm_s_	11.88	1.85
		Pg_h_-Pg_s_	14.11	3.24
		Gn_h_-Gn_s_	5.18	1.59
		Me_h_-Me_S_	4.52^ b^	1.36
Class III	33	GL_h_-GL_s_	5.75^ b^	1.48
		N_h_-N_s_	6.92^ b^	1.68
		Rhi_h_-Rhi_s_	2.94^ b^	0.87
		Ss_h_-Sn_s_	15.39^ b,c^	2.77
		U1-LS	11.83^ b^	1.63
		Inc_h_-Sto_s_	7.69^ b,c^	2.43
		L1-Li	13.81	2.15
		Sm_h_-Sm_s_	11.98	1.68
		Pg_h_-Pg_s_	15.63	2.34
		Gn_h_-Gn_s_	5.75	1.85
		Me_h_-Me_S_	5.66^ b,c^	1.51

As for vertical skeletal groups, the one-way ANOVA test showed statistically significant differences between the vertical skeletal groups (p-value≤0.05) in specific landmarks. Tukey's post hoc analysis showed that, compared to the normodivergent and hyperdivergent groups, the hypodivergent group exhibited increased facial soft tissue thickness in GL_h_-GL_s_ (p-value=0.0244), Rhi_h_-Rhi_s_ (p-value=0.0345), U1-LS (p-value=0.0513), Gn_h_-Gn_s_ (p-value=0.0118) and Me_h_-Me_s_ (p-value=0.0029), while the normodivergent showed a significant difference in Pg_h_-Pg_s_ (p-value=0.0155) (Table 5).

**Table 5:** Soft tissue thickness measurements in different vertical skeletal groups One-way ANOVA revealed differences between the three vertical groups and showed that there are significant differences in hypodivergent subjects in different landmarks: a) significant differences in hypodivergent vs. hyperdivergent (p-value≤0.05) (Tukey's post hoc test), b) significant differences in normodivergent vs. hypordivergent (p-value≤0.05) (Tukey's post hoc test), c) significant differences in normodivergent vs. hyperdivergent (p-value≤0.05) (Tukey's post hoc test). GLh-GLs: hard tissue glabella - soft tissue glabella, Nh-Ns: hard tissue nasion - soft tissue nasion, Rhih -Rhis: hard tissue rhinion - soft tissue rhinion, Ssh -Sns: hard tissue subspinale - soft tissue subnasale, U1-LS: maxillary incisor - labrale superius, Inch-Stos: hard tissue incision - soft tissue stomion, L1-Li: mandibular incisor - labrale inferius, Smh-Sms: hard tissue supramentale - soft tissue supramentale, Pgh -Pgs: hard tissue pogonion - soft tissue pogonion, Gnh -Gns: hard tissue gnathion - soft tissue gnathion, Meh -Mes: hard tissue menton - soft tissue menton

Vertical groups	N (total=170)	Variables	Mean (mm)	Standard deviation (mm)
N­­ormodivergent	85	GL_h_-GL_s_	5.36	1.27
		N_h_-N_s_	6.12	1.54
		Rhi_h_-Rhi_s_	2.66	0.78
		Ss_h_-Sn_s_	13.83	1.99
		U1-LS	10.75	2.06
		Inc_h_-Sto_s_	6.68	2.24
		L1-Li	13.50	2.14
		Sm_h_-Sm_s_	12.22	1.54
		Pg_h_-Pg_s_	15.19^ c^	2.79
		Gn_h_-Gn_s_	5.22^ b^	1.55
		Me_h_-Me_S_	4.82^ b^	1.41
Hyperdivergent	46	GL_h_-GL_s_	4.92^ a^	1.03
		N_h_-N_s_	6.07	1.14
		Rhi_h_-Rhi_s_	2.56^ a^	0.73
		Ss_h_-Sn_s_	14.19	2.35
		U1-LS	10.18^ a^	2.15
		Inc_h_-Sto_s_	6.01	2.05
		L1-Li	12.82	3.02
		Sm_h_-Sm_s_	11.76	1.87
		Pg_h_-Pg_s_	13.63^ c^	3.39
		Gn_h_-Gn_s_	4.99 ^a^	1.65
		Me_h_-Me_S_	4.44 ^a^	1.48
Hypodivergent	39	GL_h_-GL_s_	5.61^ a^	1.16
		N_h_-N_s_	6.72	1.83
		Rhi_h_-Rhi_s_	2.99^ a^	0.87
		Ss_h_-Sn_s_	14.53	2.30
		U1-LS	11.35^ a^	2.49
		Inc_h_-Sto_s_	6.88	2.46
		L1-Li	13.88	2.33
		Sm_h_-Sm_s_	11.72	1.91
		Pg_h_-Pg_s_	14.76	2.59
		Gn_h_-Gn_s_	5.99^ a,b^	1.65
		Me_h_-Me_S_	5.56^ a,b^	1.71

Furthermore, multinomial logistic regression analysis showed significant differences in terms of gender and sagittal skeletal groups (p-value≤0.05), with males less likely to be in Class II based on the references, which were female and Class I pattern. In addition, a significant association was found between age and vertical groups, specifically in the hypodivergent group (p-value≤0.05), based on the references, which were female and normodivergent (Table 6).

**Table 6:** Logistic regression model demonstrating the relationship between gender, age, and sagittal skeletal groups a - multinomial logistic regression analysis showed no significant differences between age and sagittal groups (p-value>0.05). However, the model revealed an association between gender and sagittal groups (p-value≤0.05). Our reference was the Class I group and female participants, and we found that there is a significant difference between gender in Class II where male participants were less likely to be in Class II. In Class III, however, there is no significant difference between males and females. b - multinomial logistic regression analysis showed no significant differences between gender and vertical groups (p-value>0.05). The model did, however, reveal an association between age and vertical groups (p-value≤0.05). Our reference was the normodivergent group and female participants, and the analysis showed that there is a significant difference between age and hypodivergent group.

Sagittal groups	Independent variables	Adjusted odd ratio (AOR)	95% confidence interval for AOR	p-value
Class II	Gender	0.326	(0.150, 0.712)	0.0027^a^
	Age	1.003	(0.979, 1.029)	0.7883
Class III	Gender	1.474	(0.646, 3.360)	0.3564
	Age	1.000	(0.970, 1.030)	0.9745
Hyperdivergent	Gender	0.993	(0.456, 2.166)	0.0613
	Age	1.001	(0.974, 1.028)	0.9542
Hypodivergent	Gender	2.460	(1.108, 5.458)	0.0613
	Age	1.035	(1.007, 1.065)	0.0354^b^

Based on Intraclass Correlation Coefficient (ICC) guidelines [20], the intra-examiner reliability of all soft tissue measurements was good (ICC values were between 0.75 and 0.90).

## Discussion

Facial esthetics is the principal goal in orthodontic practice and the main motivation for patients seeking orthodontic treatment [5, 21]. Orthodontists can identify skeletal patterns associated with unpleasant soft tissue thickness and transform it into attractive facial thickness by means of orthodontic treatment, orthognathic surgery, or plastic surgery. This study used CBCT images to assess the facial soft tissue thickness in different sagittal and vertical skeletal patterns. A combined assessment of both vertical and sagittal skeletal patterns has not previously been undertaken in the Saudi Arabian population. A previous study conducted on Saudi Arabian subjects assessed facial soft tissue differences arising from different dental malocclusions instead of skeletal discrepancies [22].

Earlier studies have shown that changes in the skeletal structures are reflected in the overlying soft tissue. For example, maxillary and dental protrusion affects the soft tissue protrusion and upper lip position [23, 24]. Moreover, alterations in facial soft tissue have been reported after orthognathic surgery [25]. Hence, the present study excluded subjects with previous orthodontic treatment and orthognathic surgeries. In addition, subjects with previous plastic surgeries were excluded. Furthermore, CBCT images of patients with difficulty in lip closure due to severe skeletal discrepancy were excluded from the study.

Our findings demonstrated significantly greater soft tissue thickness in all landmarks in male subjects compared to female subjects, except for the Sm_h_-Sm_s_ landmark. This result is in line with previous studies that were conducted in different populations [7, 9, 17, 26-28], suggesting the presence of sexual dimorphism in facial soft tissue thickness. Males have thicker facial bones and muscles compared to females, and that could explain the gender differences in facial soft tissue [29]. In addition, males' skin is thicker since the testosterone hormone promotes collagen synthesis, unlike in females and in whom the estrogen hormone promotes hyaluronic acid synthesis [30].

With respect to the skeletal sagittal groups, the initial hypothesis was rejected, indicating that there were some statistically significant differences between the groups. Specifically, the soft tissue thickness was increased in GL_h_-GL_s_, N_h_-N_s_, Rhi_h_-Rhi_s_, Ss_h_-Sn_s_, U1-LS, Inc_h_-Sto_s_, and Me_h_-Me_s_ landmarks in the skeletal Class III group compared to Class I and Class II. In agreement with our results, previous studies reported thicker soft tissue in the maxilla in class III subjects [9, 26, 27]. The thicker soft tissue in skeletal class III in our studied population can be explained by soft tissue compensation in skeletal regions with deficient skeletal development or positioned posteriorly. In addition, the upper incisors were proclined, and lower incisors were retroclined in the majority of class III individuals, and this could push the upper lip forward, leading to greater soft tissue thickness [9].

The influence of the vertical skeletal pattern on facial soft tissue thickness has been reported previously in other populations [31-33]. In agreement with our results, a study showed that the hypodivergent group had thicker soft tissue in Me_h_-Me_s_ and Gn_h_-Gn_s_ [34]. Previous studies found that hyperdivergent subjects had reduced soft tissue thickness in Rhi_h_-Rhi_s_, Ss_h_-Sn_s_, and U1-LS, compared to hypodivergent subjects [32, 33]. In concordance with previous literature, our findings highlight that the hyperdivergent group had reduced soft tissue thickness in GL_h_-GL_s_, Rhi_h_-Rhi_s_, U1-LS, Gn_h_-Gn_s_, and Me_h_-Me_s_ compared to the hypodivergent group. This could be explained by soft tissue compensation for the skeletal vertical disharmony [22]. Another reason could be due to the increased stretching of the soft tissue in the hyperdivergent group, resulting in reduced thickness. On the other hand, the hypodivergent group had increased soft tissue thickness, probably due to the presence of hypertrophy of perioral musculature, which tends to occur as a way of compensating for vertical deficiency and maintaining lip competence [35].

Although the results of this study highlight that male subjects are more likely to be in skeletal class III in relation to female subjects, our analysis showed no significant differences between gender and vertical groups. This can be explained by the late onset of the pubertal growth spurt and increased pubertal growth duration in males compared to females, resulting in continued mandibular size growth leading towards a skeletal class III relationship [36]. In agreement with our findings, a study reported class III tendency in male subjects. However, this study reported a significant difference between vertical groups and gender. Male subjects were more likely to be hypodivergent, and hyperdivergence was found in female subjects [37]. It is worth noting that the study used different measurement tools (anteroposterior dysplasia indicator (APDI), and sum total of nasion-sella-articulaire angle, sella-articulaire-gonion angle, and articulaire-gonion-mention angle (sum angle)) for grouping the subjects into sagittal and vertical patterns instead of ANB and FMA angles. Thus, the disagreement in findings could be the different measurement tools and different ethnic backgrounds.

Our results support previous studies [38-40] that age-related changes of the face are associated with hypodivergence of the face. The mandibular plane decreases with age, and the posterior-to-anterior facial height ratio increases with age, possibly due to the counterclockwise rotation of the mandible with age. In addition, similar to the results of previous studies, our data reported high intra-examiner reliability, suggesting that CBCT scans are reliable for facial soft tissue analysis [9, 41].

The clinical implication of the present study includes providing a diagnostic tool offering a reliable assessment for the diagnosis and treatment of different skeletal patterns. Orthodontists and surgeons should be familiar with sex-specific variations in certain populations during therapeutic interventions. Considering the studied population, the presence of facial soft tissue sexual dimorphism in adults has important implications in orthodontic treatment planning and outcome assessment. A study found that patients with thick lips displayed no correlation between incisor retraction and lip retraction, whereas patients with reduced lip thickness showed a significant correlation [42]. Based on this information and our study findings, orthodontists should be mindful that extraction therapy and incisor retraction might have a greater effect on facial profiles in female patients compared to male patients. Moreover, the present study indicates different clinical considerations for different skeletal patterns. Patients with skeletal class III and hypodivergent patterns might be less affected by orthodontic extraction treatment and incisor retraction compared to other skeletal patterns.

Although this study is the first of its nature in Saudi Arabia, there were some limitations, such as the small sample size. Moreover, since it is a retrospective study, there was a lack of information regarding the body mass index (BMI). Future studies with larger sample sizes are therefore recommended in order to obtain information about the body mass index. 

## Conclusions

The facial soft tissue of Saudi Arabian adults in different sagittal and vertical skeletal relationships was analyzed by using 3D CBCT images. When we compared the values between genders, sexual dimorphism was marked in facial soft tissue measurements, in which male participants have increased facial soft tissue thickness in most landmarks compared to female participants. When comparing the soft tissue measurements between sagittal and vertical skeletal patterns, the results of the study highlighted that skeletal Class III and hypodivergent groups have been shown to have thicker soft tissue in specific facial soft tissue landmarks. Moreover, this study demonstrates that male subjects were more likely to be in skeletal class III in relation to female subjects. The present study provides a basis for a diagnostic tool for a reliable assessment and treatment of different skeletal patterns.
